# The First Transmembrane Domain of Lipid Phosphatase SAC1 Promotes Golgi Localization

**DOI:** 10.1371/journal.pone.0071112

**Published:** 2013-08-01

**Authors:** Jinzhi Wang, Juxing Chen, Caroline A. Enns, Peter Mayinger

**Affiliations:** 1 Division of Nephrology & Hypertension, Oregon Health & Science University, Portland, Oregon, United States of America; 2 The Department of Cell & Developmental Biology, Oregon Health & Science University, Portland, Oregon, United States of America; Institute of Biology Valrose, France

## Abstract

The lipid phosphatase Sac1 cycles between endoplasmic reticulum and cisternal Golgi compartments. In proliferating mammalian cells, a canonical dilysine motif at the C-terminus of Sac1 is required for coatomer complex-I (COP-I)-binding and continuous retrieval to the ER. Starvation triggers accumulation of Sac1 at the Golgi. The mechanism responsible for Golgi retention of Sac1 is unknown. Here we show that the first of the two transmembrane regions in human SAC1 (TM1) functions in Golgi localization. A minimal construct containing only TM1 and the adjacent flanking sequences is concentrated at the Golgi. Transplanting TM1 into transferrin receptor 2 (TfR2) induces Golgi accumulation of this normally plasma membrane and endosomal protein, indicating that TM1 is sufficient for Golgi localization. In addition, we determined that the N-terminal cytoplasmic domain of SAC1 also promotes Golgi localization, even when TM1 is mutated or absent. We conclude that the distribution of SAC1 within the Golgi is controlled via both passive membrane thickness-dependent partitioning of TM1 and a retention mechanism that requires the N-terminal cytoplasmic region.

## Introduction

The lipid phosphatase Sac1 is an evolutionary conserved regulator of phosphatidylinositol-4-phosphate (PtdIns(4)P) and plays vital roles at endoplasmic reticulum (ER)/plasma membrane contact sites and in Golgi organization and trafficking [1]-[3]. Sac1 is an integral membrane protein containing a large N-terminal cytoplasmic domain and two transmembrane regions near its C terminus [4]. The crystal structure of the N-terminal catalytic domain of yeast Sac1p was resolved [5], but the arrangement of the two transmembrane regions and their role in Sac1p localization has not been determined. In mammals, Sac1 cycles continuously between ER and Golgi compartments via the canonical trafficking mechanisms involving coat protein complex-I (COP-I) and coat protein complex-II (COP-II) [6]. In proliferating cells, Sac1 localizes to both ER and cisternal Golgi regions but is absent from the trans-Golgi network (TGN) [7]. The polarized distribution of Sac1 is critical for maintaining a steep PtdIns(4)P gradient at the Golgi complex with the majority of PtdIns(4)P confined to the TGN [7]. During starvation and in quiescent cells Sac1 accumulates at the TGN, which reduces Golgi PtdIns(4)P levels and slows anterograde trafficking [1]. All mammalian Sac1 orthologs contain two distinct sequence motifs that are required for shuttling between ER and Golgi. A dilysine motif at the C terminus of Sac1 is essential for interaction with the COP-I machinery and for retrieval to the ER [1], [8]. A leucine repeat region at the cytoplasmic N-terminal domain promotes oligomerization of Sac1 during starvation, which is a prerequisite for Golgi accumulation [1]. However, how the distribution of Sac1 within the Golgi is controlled remains unclear.

The Golgi apparatus is a central sorting organelle guiding soluble and membrane-bound proteins arriving from the ER to their different endomembrane compartments. Passage through the Golgi is also required for post-translational processing of secretory proteins [9]. The mechanisms responsible for the characteristic intra-Golgi distribution of resident enzymes that are required for glycosylation and other modifications are not well established. Unlike the classic retrieval motifs that control steady state localization of ER proteins, no common Golgi sorting sequence has been found [9], [10]. Two mechanisms have been proposed for Golgi retention, commonly referred to as the oligomerization and the bilayer-thickness model [9], [11]. The former proposes that retention of Golgi enzymes is achieved by forming large oligomers, which prevent sorting into anterograde transport vesicles [12], [13]. The latter postulates that retention relies on the length of membrane-spanning domains and the membrane bilayer thickness of the individual Golgi sections [12].

In this study, we determined that two distinct regions within human SAC1 function in Golgi retention. We found that a minimal construct containing only the first of the two transmembrane segments of SAC1 (TM1) is efficiently concentrated at the Golgi. In addition, the large N-terminal cytosolic region of SAC1 was independently sufficient for Golgi localization. These results suggest that Golgi distribution of SAC1 is controlled via both membrane domain-dependent partitioning and protein-protein interactions.

## Materials and Methods

### Cells and Antibodies

HeLa cells were cultured in Dulbecco’s modified Eagle medium containing 10% fetal bovine serum (Thermo scientific), 1 mM glutamine, 1 mM sodium pyruvate (Invitrogen). Cells were incubated in 5% CO_2_ humidified incubator at 37°C. Anti-GM130 mouse mAb was purchased from BD Biosciences (catalog number: 610822). Anti-GRASP65 rabbit polyclonal serum was purchased from Novus Biological (catalog number: NBP1-57592). Anti-Sec61β polyclonal IgG was obtained from Santa Cruz (catalog number: SC-27695). Anti-flag M2 mouse mAb was purchased from Sigma (catalog number: F1804). Alexa Fluor 488-conjugated goat anti-rabbit IgG and Alexa Fluor 546-conjugated goat anti-mouse IgG were purchased from Molecular Probes (catalog numbers: A-11008 and A-21143). Fluorescently labeled wheat germ agglutinate, WGA647, was obtained from Invitrogen (catalog number; W32466).

### Plasmids

Plasmids pGFP-SAC1 and pGFP-SAC1-K2A were described previously [1]. To generate pGFP-SAC1(478–549) and pGFP-SAC1(478–587)-K2A constructs, corresponding cDNAs were amplified by PCR and digested with BamHI and SalI, ligated into pEGFP-C1 vector digested with BglII and SalI by utilizing the isoschizomers BamHI and BglII. To construct pGFP-SAC1(501–549) and pGFP-SAC1(512–549), cDNA fragments encoding GFP-SAC1(512–549) and GFP-SAC1(501–549) were amplified by PCR using pGFP-SAC1(512–587) or pGFP-SAC1(501–587) as templates respectively [1]. The PCR products were digested with BamHI and SalI and subcloned into vector pCMV-3Tag-1A. To generate the pGFP-TfR2(73–801) construct, human transferrin receptor 2 (TfR2) cDNA encoding amino acid 73–801 was amplified by PCR and cloned into expression vector pEGFP-C2 by utilizing EcoRI and SalI restriction sites.

The SAC1 chimeric constructs containing the transmembrane domain of TfR2 (TMtfr2), pGFP-SAC1(TMtfr2)-K2A and pGFP-SAC1(478–549)TMtfr2, were generated by overlap extension PCR. The nucleotide sequence encoding TM1 of SAC1 was replaced with the sequence encoding the entire 24-residue TMtfr2. The PCR fragments were digested with BamHI and SalI and ligated into pEGFP-C1 as described above. Similarly, a TfR2 chimera containing the TM1 region of SAC1, GFP-TfR2(73–801)TM1, was generated by overlap extension PCR. The nucleotide sequence encoding TMtfr2 was substituted with the sequence encoding the entire 23-residue TM1 of SAC1. The cDNA was digested with EcoRI and SalI and ligated into pEGFP-C2 as described above.

To construct pFlag-SAC1(1–549) and pFlag-SAC1(1–549)TMtfr2, corresponding cDNA fragments were amplified by PCR using GFP-SAC1 and GFP-SAC1(TMtfr2)-K2A as templates respectively, and then subcloned into vector pCMV-3Tag-1A with BamHI and SalI. To construct pGFP-SAC1(152–549)TMtfr2 and pGFP-SAC1(152–549), pFlag-SAC1(1–549)TMtfr2 and pFlag-SAC1(1–549) were digested with EcoRI and SalI and the resulting fragments of SAC1 were subcloned into vector pEGFP-C2 by utilizing the same restriction sites. All constructs were verified by sequencing. (GenBank accession numbers: TfR2, BC142630.1; SAC1, NM014016.3).

### Mutagenesis

The plasmid pGFP-SAC1(1–518) was constructed by introducing two adjacent stop codons by site-directed mutagenesis using pGFP-SAC1 as a template. pGFP-SAC1(478–549)-E2A, pGFP-SAC1(478–549)C2S, pGFP-SAC1(478–549)ins3L, pGFP-SAC1-K2Ains3L were generated by mutagenesis using primers containing the desired mutations. QuikChange II XL Site-Directed Mutagenesis Kit was purchased from Agilent Technologies (catalog number: 200521). Primers were designed by utilizing online Quikchange Primer Design Software (https://www.genomics.agilent.com/). Experiments were performed according to manufacturer’s instructions.

### Immunofluorescence and Confocal Microscopy

Transient transfections were performed using the lipofectamine2000 transfection reagent (Invitrogen) according to the manufacturer’s protocol. For confocal microscopy, 1.5×10^5^ HeLa cells were plated into each well of a 6-well plate (Corning) containing glass coverslips and allowed to attach for 18–20 h at 37°C. The cells were transfected with 1 µg of DNA mixed with 3 µl lipofectamine2000 and incubated for 24 h at 37°C.

24-hour after transfection, HeLa cells were washed three times with PBS and fixed with 4% paraformaldehyde in PBS for 15 min. Cells were treated with blocking & permeabilization buffer (PBS, 2% normal goat serum, 0.5% Triton X-100) for 30 min. The cells were then incubated with antibody diluted in PBS for 1 h. Primary antibodies used were anti-GM130 mouse mAb (2.5µg/ml final concentration), anti-flag M2 mouse mAb (1∶500) and anti-GRASP65 rabbit polyclonal sera (1∶200). Cells were washed four times with washing buffer (PBS, 0.1% Triton X-100, 0.2% BSA) and incubated with secondary antibody Alexa Fluor 488-conjugated goat anti-rabbit IgG (1∶500) or Alexa Fluor 546-conjugated goat anti-mouse IgG (1∶500). Cells were then washed again four times with washing buffer. For cell surface staining, fixed cells were incubated with 5.0 µg/mL of WGA647 conjugate for 20 minutes at room temperature followed by two washes. Coverslips were mounted onto microscope slides using Fluoromount-G (SouthernBiotech) and visualized on a Zeiss LSM 700 laser-scanning confocal microscope with a 63×1.4 NA Plan-Apochromat objective lens. Excitation wavelengths of 488 nm (GFP; Alexa-488) or 555 nm (Alexa-546) were used. Images were analyzed using ZEN 2009 software. The depicted images are typical representatives of cells expressing a particular construct. All localization experiments were repeated at least twice.

## Results

### Identification of Golgi Localization Domains in SAC1

**Figure 1:**
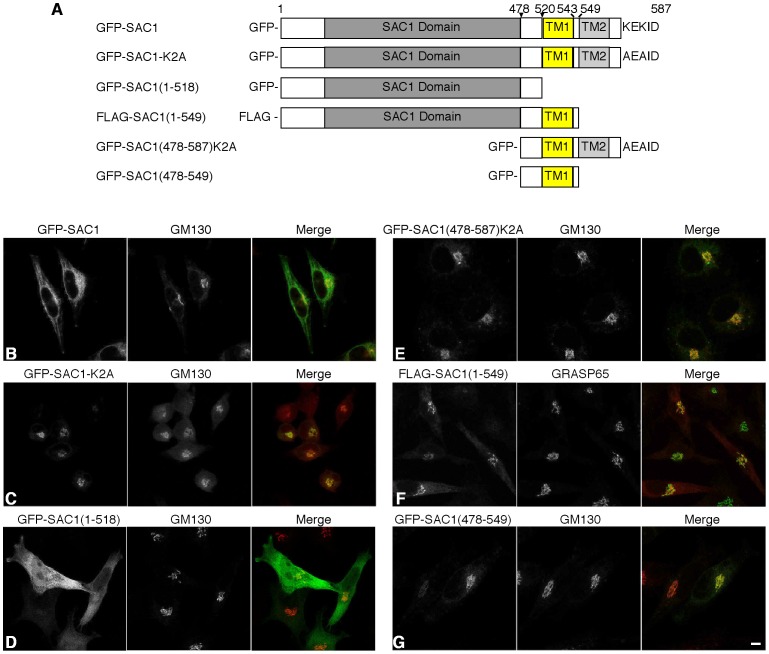
Mapping the Golgi retention motif in SAC1. (**A**) Overview of truncated SAC1 constructs. (**B–G**) HeLa cells were transfected with the indicated FLAG-tagged (red) or GFP-tagged (green) SAC1 constructs, costained with anti-GM130 (red) or anti-GRASP65 antibodies (green) and analyzed by confocal immunofluorescence microscopy. (**B**) GFP-SAC1; (**C**) GFP-SAC1-K2A; (**D**) GFP-SAC1(1–518); (**E**) GFP-SAC1(478–587)-K2A; (**F**) FLAG-SAC1(1–549); (**G**) GFP-SAC1(478–549). Scale bar, 50 µm.

The human phosphoinositide phosphatase SAC1 is an integral membrane protein with two transmembrane domains near the C-terminus (Figure 1A) [4], [14]. Consistent with our previous studies [1], transiently expressed GFP-SAC1 is mainly localized at the ER but also shows colocalization with the Golgi marker GM130 (Figure 1B). In contrast, GFP-SAC1-K2A, in which the two lysines in the C-terminal retrieval motif were replaced by alanines, predominately accumulates at the Golgi apparatus [1], (Figure 1C). To map the regions in SAC1 that are required for Golgi targeting, a series of GFP and flag-tagged truncation mutants was constructed (Figure 1A). The intracellular localization of these SAC1 mutants was analyzed by confocal microscopy and compared with either endogenous GM130 or endogenous GRASP65 as Golgi markers. To examine if the large cytoplasmic N-terminal domain contains a Golgi retention signal, GFP-SAC1(1–518) was constructed, in which the entire cytoplasmic N-terminal domain was fused to GFP. GFP-SAC1(1–518) displayed cytoplasmic distribution but also showed some localization at the Golgi (Figure 1D), suggesting that the N-terminal domain itself may have a low affinity for the Golgi. However, this domain was not required for efficient Golgi retention because the mutant GFP-SAC1(478–587)-K2A, in which most of the cytosolic N-terminal domain was deleted, colocalized with the Golgi marker GM130 (Figure 1E). Furthermore, the truncation mutant flag-SAC1(1–549), which contains the entire cytoplasmic N-terminal domain, the transmembrane domain TM1, and six amino acids predicted to face the lumen, also showed Golgi localization (Figure 1F). These results suggested that the region from amino acid 478 to 549, which contains TM1 and short flanking sequences, was sufficient for Golgi retention. To test this idea, the GFP-tagged construct GFP-SAC1(478–549), containing only this region, was generated. Immunofluorescence microscopy showed that GFP-SAC1(478–549) displayed accumulation at the Golgi (Figure 1G). Whereas wild-type SAC1 shows a characteristic distribution at both ER and Golgi regions [1], a large proportion of cells expressing GFP-SAC1(478–549) showed the GFP signal confined to the Golgi (83% ±3%; mean ± SD from two experiments, 50 cells each). Together these data indicate that the short GFP-SAC1(478–549) construct contains all the elements that are needed for efficient Golgi retention.

### Elimination of the Cytoplasmic Region Adjacent to TM1 in GFP-SAC1(478–549) causes Accumulation in the ER

**Figure 2:**
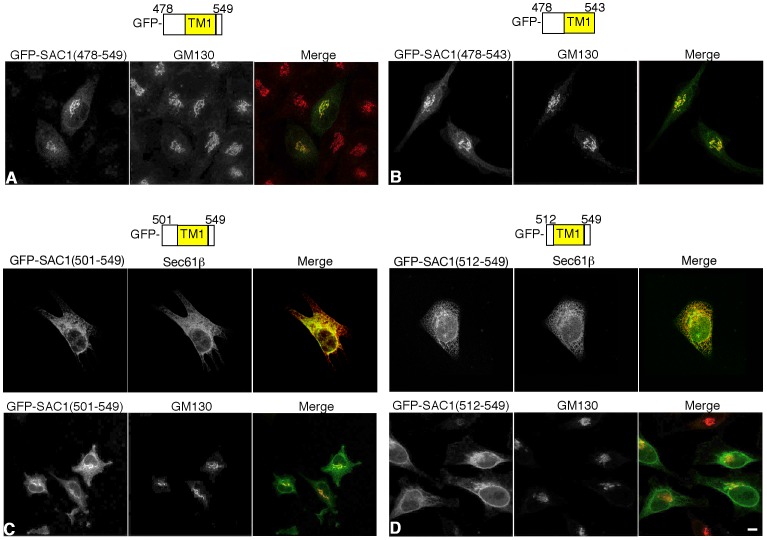
The cytoplasmic flanking region of TM1 is required for ER export. HeLa cells were transfected with the indicated GFP-tagged SAC1 constructs (green), costained with anti-GM130 antibodies (red) or Sec61β (red) and analyzed by confocal immunofluorescence microscopy. (**A**) SAC1(478–549); (**B**) GFP-SAC1(478–543); (**C**) GFP-SAC1(501–549); (**D**) GFP-SAC1(512–549). Scale bar, 50 µm.

GFP-SAC1(478–549) contains a short 43 amino acid stretch that is part of the N-terminal cytoplasmic domain and six amino acids at the C-terminus that are predicted to face the ER and Golgi lumen. To further narrow down the sequence required for Golgi retention, a construct GFP-SAC1(478–543) was generated, in which the potential luminal region 544-DTWTET-549 was removed. GFP-SAC1(478–543) showed the same strong co-localization with Golgi marker GM130 as GFP-SAC1(478–549) (Figure 2A,B), suggesting that this short stretch in the SAC1 sequence is not required for Golgi retention. To examine whether the adjoining N-terminal cytoplasmic region adjacent to TM1 is required for Golgi retention, pGFP-SAC1(501–549) and pGFP-SAC1(512–549) were constructed. Fluorescence microscopy showed that GFP-SAC1(501–549) localized at both the Golgi and the ER (Figure 2C). In contrast, GFP-SAC1(512–549) was largely restricted to the ER compartment, where it colocalized with Sec61β (Figure 2D). This result suggested that elimination of cytoplasmic region that flanks TM1 causes retention at the ER. This region contains the sequence 505-DELE-508, which resembles the classical COP-II binding motif DXE [15]. However, a GFP-SAC1(478–549)-E2A mutant showed no ER retention (Figure S1) and it remains unclear whether and how this portion of the protein is required for SAC1 trafficking.

### The TM1 Segment of SAC1 is Sufficient for Golgi Targeting

**Figure 3:**
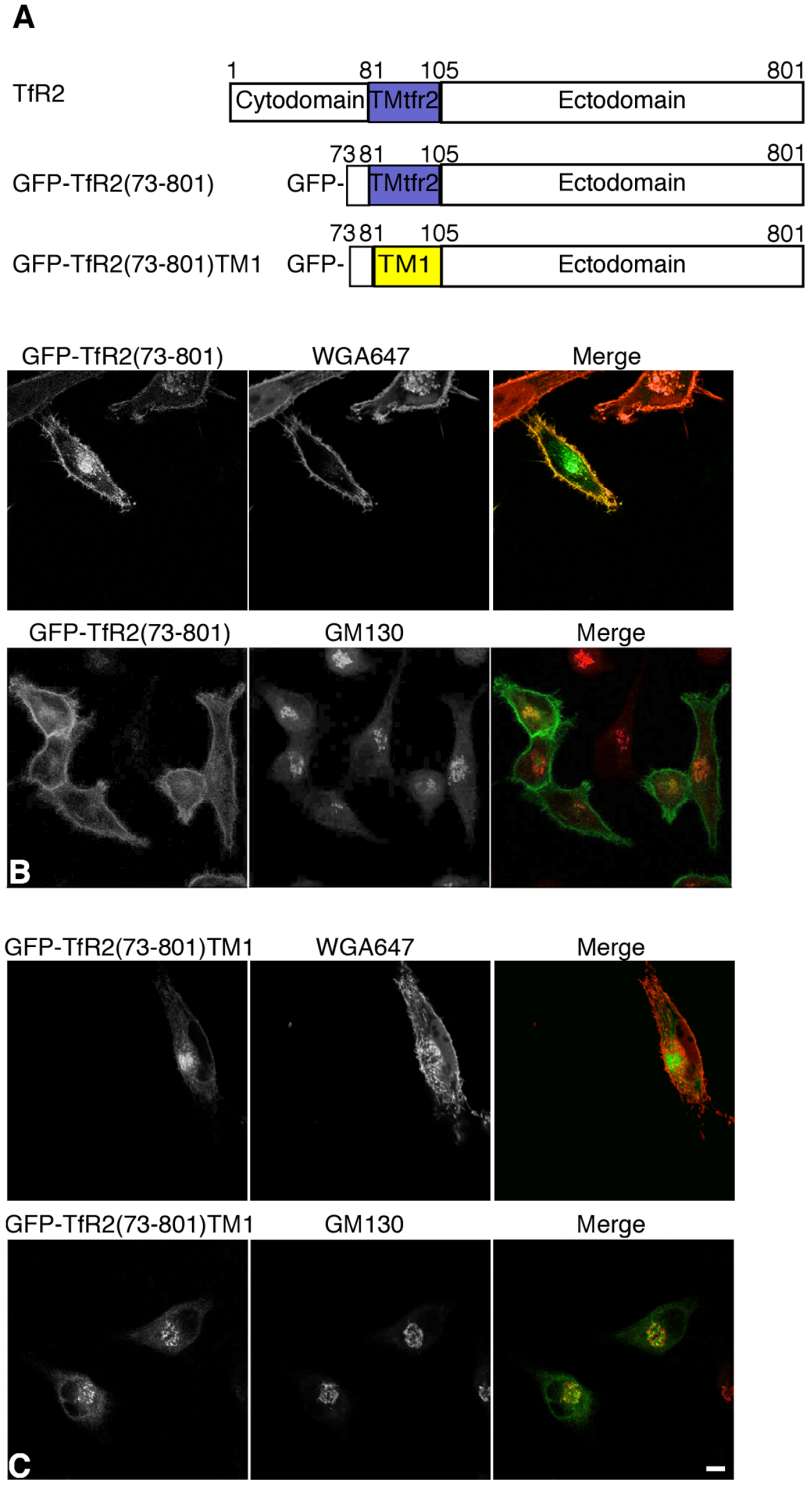
Insertion of TM1 induces Golgi retention of TfR2. HeLa cells were transfected with the indicated GFP-tagged TfR2 constructs that lack the N-terminal endocytic sorting motif (green), costained with anti-GM130 antibodies (red) or WGA647 (red) and analyzed by confocal immunofluorescence microscopy. (**A**) GFP-TfR2(73–801); (**B**) GFP-TfR2(73–801)TM1. Scale bar, 50 µm.

In order to determine if the TM1 of SAC1 is sufficient for promoting Golgi association, we replaced the transmembrane domain of TfR2 with TM1. TfR2 is an 801-residue type II membrane protein with a small cytoplasmic domain (amino acids 1–80), a putative 24-residue TM domain (amino acids 81–104) and a large ectodomain (amino acids 105–801). At steady state, wild-type TfR2 localizes mainly to the plasma membrane and intracellular endosomes [16], [17]. To achieve a clear and distinguishing localization phenotype, a GFP-TfR2(73–801) mutant was generated in which most of the cytoplasmic domain of TfR2 containing the YQRV endocytic motif was removed. The corresponding chimera GFP-TfR2(73–801)TM1 was also constructed (Figure 3A). GFP-TfR2(73–801) showed plasma membrane localization in transfected cells and colocalized with fluorescently labeled weat germ agglutinin (WGA647) when analyzed by fluorescence microscopy (Figure 3B). In contrast, GFP-TfR2(73–801)TM1 was retained predominantly at the Golgi and no cell surface staining was detectable (Figure 3C). Furthermore, replacing TM1 in GFP-SAC1(478–549) with the transmembrane domain of TfR2 impaired Golgi retention (Figure S2). These data suggest that the TM1 region of SAC1 is sufficient to promote Golgi localization.

### Lengthening of TM1 Triggers Plasma Membrane Accumulation of GFP-SAC1(478–549)

**Figure 4:**
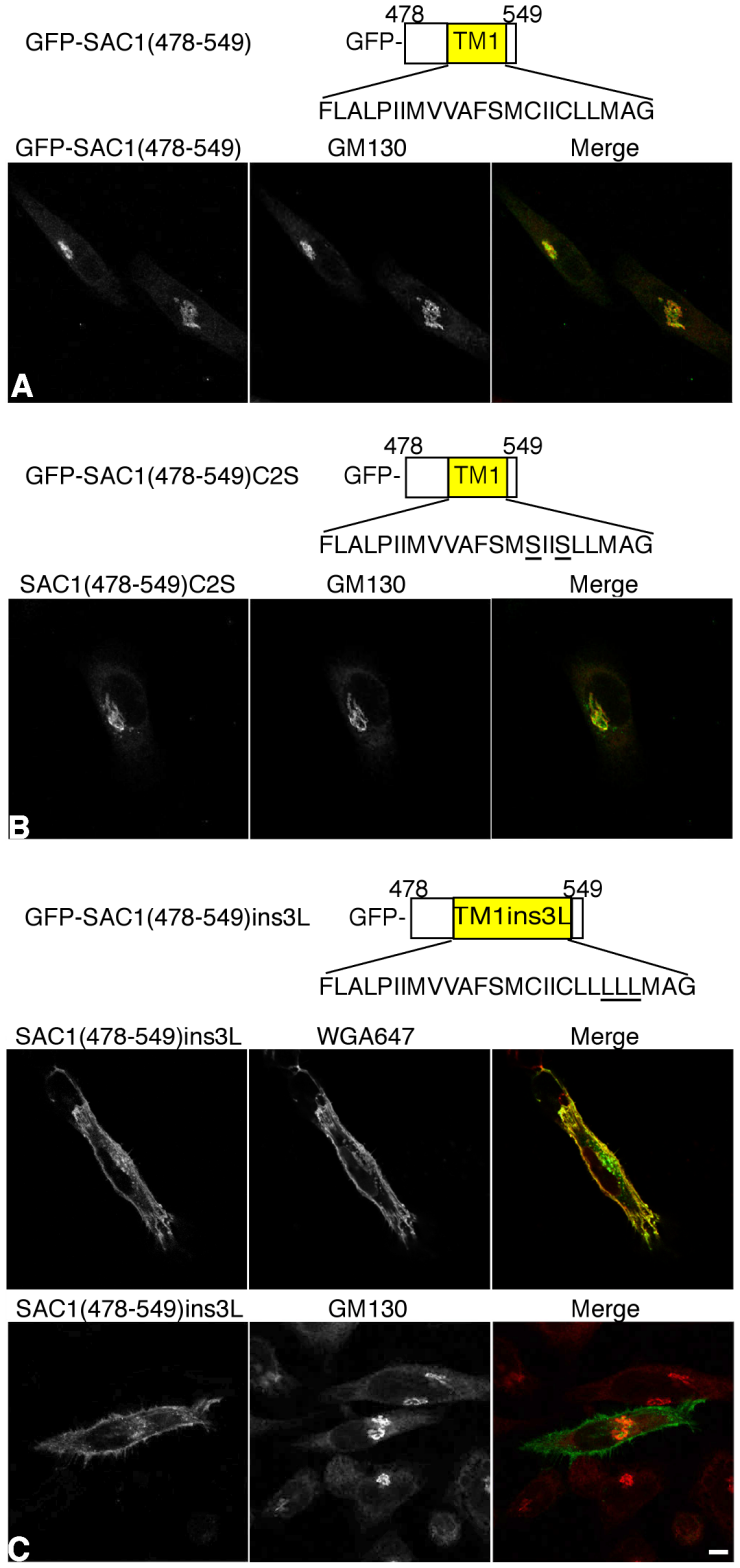
Lengthening TM1 by inserting three leucine residues results in a loss of Golgi retention. HeLa cells were transfected with the indicated GFP-tagged SAC1 constructs (green), costained with anti-GM130 antibodies (red) or WGA647 (red) and analyzed by confocal immunofluorescence microscopy. (**A**) GFP-SAC1(478–549); (**B**) GFP-SAC1(478–549)C2S; (**C**) GFP-SAC1(478–549)ins3L. Scale bar, 50 µm.

Sequence alignments of the membrane-spanning domains of resident Golgi proteins show no sequence homology and no particular motif required for retention has been identified [18], [19]. However, in several Golgi enzymes cysteine residues within transmembrane domains play a role in Golgi retention or in oligomerization [20]-[23]. There are two cysteine residues present within TM1 of human SAC1 (FLALPIIMVVAFSMCIICLLMAG). To test whether these residues are critical for Golgi retention, they were mutated to serines. The corresponding GFP-SAC1(478–549)C2S mutant showed the same strong colocalization with Golgi marker GM130 as the unsubstituted GFP-SAC1(478–549) and no cell surface labeling was detected (Figure 4A,B), demonstrating that the cysteines are not critical for Golgi localization.

According to the bilayer thickness model for Golgi retention, the length of transmembrane segments in resident Golgi proteins is the key determinant for localization [11], [12]. To examine if increasing the length of TM1 interferes with its capability to promote Golgi localization, three leucine residues were inserted into this region converting it into a 26-residue-long hydrophobic domain (FLALPIIMVVAFSMCIICLLLLLMAG, inserted leucines are underlined). The elongated TM1 mutant, GFP-SAC1(478–549)ins3L, showed accumulation at the plasma membrane and reduced Golgi staining (Figure 4C). These data further support the idea that TM1 functions in the localization of SAC1 and indicate that the length of this domain is critical for proper Golgi partitioning.

### The N-terminal Cytoplasmic Domain of SAC1 Contributes Golgi Localization

**Figure 5:**
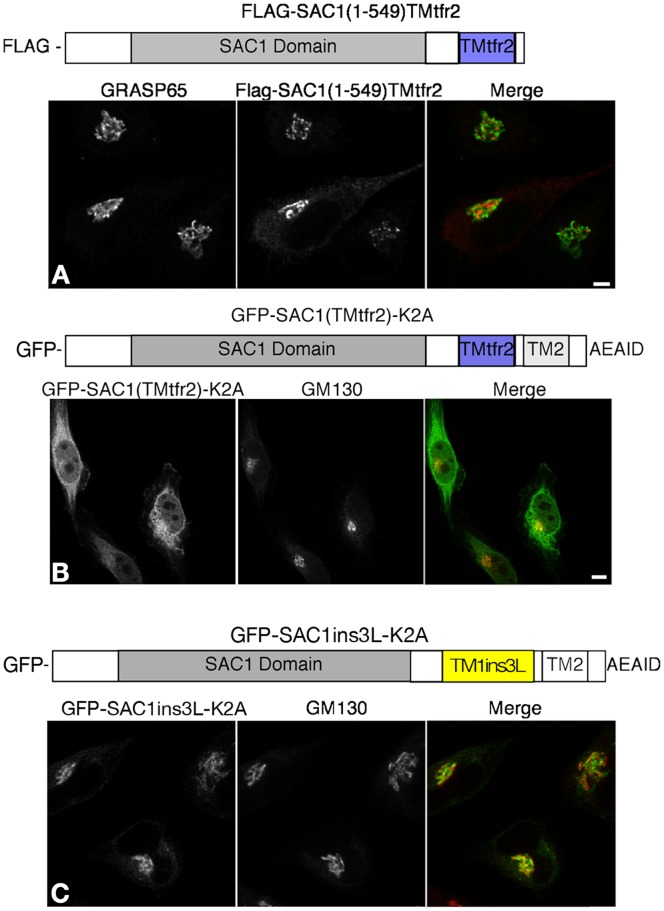
N-terminal domain of SAC1 promotes Golgi retention. HeLa cells were transfected with the indicated SAC1 constructs, costained with anti-GM130 antibodies (red) and analyzed by confocal immunofluorescence microscopy. (**A**) flag-SAC1(1–549)TMtfr2; (**B**) GFP-SAC1(TMtfr2)-K2A; *C*, GFP-SAC1ins3L-K2A. Scale bar, 50 µm.

Because a truncated version of Sac1 comprising only the N-terminal cytoplasmic domain showed partial colocalization with Golgi markers (Figure 1D), we reasoned that this portion of the protein might independently promote Golgi targeting. To test this idea, we generated a construct in which the entire N-terminal region of Sac1 was fused to TMtfr2. The Flag-SAC1(1–549)TMtfr2 chimera efficiently accumulated at the Golgi and no plasma membrane localization was observed (Figure 5A). This result therefore suggested that the N-terminal region of Sac1 also functions in Golgi targeting in addition to the TM1-specific mechanism. Based on the structure of yeast Sac1p, the N-terminal region is comprised of three distinct domains, the SacN domain (1–186), the catalytic domain (187–462) and an unstructured region (462–521). Our attempts to analyze these individual domains for their potential roles in Golgi targeting were unsuccessful because the relevant truncated constructs GFP-SAC1(152–549) and GFP-SAC1(152–549)TMtfr2 formed aggregates and we were therefore unable to perform localization analyses (data not shown).

To examine the relevance of TM1 in the context of full-length SAC1, we replaced TM1 with either TMtfr2 or with the elongated TM1 that contains three additional leucines. The GFP-SAC1(TMtfr2)-K2A protein accumulated in the ER and was not present at Golgi membranes (Figure 5B). In contrast, the mutant GFP-SAC1ins3L-K2A localized to the Golgi where it was efficiently retained because no escape to the plasma membrane could be observed (Figure 5C). The TM1 region of SAC1 is therefore not strictly required for Golgi localization of the full-length SAC1 protein that lacks the COP-I retrieval motif. However, TM1 may play additional roles in the proper folding and arrangement of the two transmembrane segments, which is a prerequisite for ER exit.

## Discussion

Mammalian Sac1 orthologs have several unique properties related to their tightly controlled intracellular localization. In proliferating cells, Sac1 displays a specific distribution between the ER and the cis-Golgi, which is essential for spatial control of PtdIns(4)P [7]. Serum starvation induces quantitative accumulation of Sac1 at the Golgi but this translocation is promptly reversed upon growth factor stimulation [1]. Previous studies have shown that a canonical C-terminal dilysine motif, present in all mammalian Sac1 orthologues, is required for steady state distribution at the ER [1], [8]. In this report, we show that one of the two transmembrane domains in human SAC1 is sufficient for partitioning into the Golgi. The relevance of a transmembrane segment for Golgi localization is not unprecedented. For example, the first of three membrane-spanning domains of E1 glycoprotein of avian coronavirus was found to be sufficient to retain this protein in the cis-Golgi [24]. Furthermore, the single transmembrane domains in many Golgi glycosylation enzymes are key determinants of their specific localization at the Golgi [8]. How transmembrane domains mediate Golgi retention is not entirely clear and two mechanisms have been proposed, which are based either on oligomerization of Golgi enzymes into large complexes or on membrane thickness. SAC1 can form oligomers and oligomerization promoted by starvation coincides with Golgi accumulation [1]. However, the data presented here show that a minimal construct encompassing only transmembrane region TM1 plus short flanking regions efficiently accumulates at the Golgi. Furthermore, transplanting the TM1 sequence without its flanking regions into TfR2 was sufficient to redirect this protein to the Golgi. Our experiments also show that lengthening of TM1 significantly reduced Golgi retention and induced plasma membrane localization. Based on these results we propose that bilayer thickness may contribute to the precise distribution of Sac1 within the Golgi.

Based on the bilayer thickness model, relatively short transmembrane domains are thought to correlate with Golgi retention [25], [26]. A recent bioinformatic investigation used comprehensive comparisons of transmembrane domains of integral membrane proteins to show that the transmembrane length of resident membrane proteins varies along the secretory pathway [27]. The mean hydrophobic length of transmembrane domains in vertebrate ER and Golgi proteins is 20.6 residues, whereas TGN membrane proteins have a mean value of 24.8 hydrophobic residues [27]. The predicted length of TM1 is 23 amino acids, which is longer than the common transmembrane spanning domains of Golgi enzymes [27]. It is therefore tempting to speculate that the specific dimensions of TM1 allow SAC1 to reach the TGN, which may be physiologically important because the phosphatase is responsible for downregulation of TGN PtdIns(4)P levels during starvation.

A specific distribution of human SAC1 within the Golgi is required to establish a cis-to-trans PtdIns(4)P gradient in proliferating cells [1], [7]. The N-terminal cytoplasmic region of SAC1 may play multiple roles in this process. Oligomerization of this domain is a key determinant in controlling ER exit or interaction with COP-I because oligomerization-deficient SAC1 mutants remain concentrated at the ER during starvation [1]. The presented data suggest that the N-terminal region of SAC1 plays an additional role in the targeting of SAC1 to the Golgi. In yeast, the N-terminal domain of Sac1p interacts with the peripheral Golgi protein Vps74, a PtdIns(4)P effector required for Golgi localization of glycosyltransfreases [28]. Whether GOLPH3, the human homolog of Vps74 [29], interacts with SAC1 has not been examined. However, deletion of Vps74 in yeast does not abrogate Golgi localization of Sac1p and there is evidence that additional integral Golgi membrane proteins are involved in Sac1p localization [30].

In summary, we have identified two specific regions in human SAC1 that are independently involved in Golgi targeting. Combined with our previous studies, these results support a multi-step mechanism controlling distribution of SAC1 at the Golgi. During proliferation, SAC1 partitions into the cisternal Golgi but is continuously retrieved to the ER by interacting with retrograde COP-I complexes [1], [7]. The first transmembrane segment TM1 is sufficient to bring about Golgi localization but only if the COP-I sorting motif is absent. Because Golgi partitioning via transmembrane domains is a passive process an additional retention mechanism must be in place to establish the steady state distribution of SAC1 at the cisternal Golgi in proliferating cells. Our data show that the N-terminal domain of SAC1 plays an important role in this process. This view is supported by our previous work showing that elimination of the N-terminal domain of SAC1 triggers ER accumulation if the C-terminal COP-I binding motif is intact [1]. When ER retrieval is turned off during starvation, SAC1 behaves like a resident Golgi enzyme and TM1 may then direct the protein to Golgi regions with appropriate membrane thickness resulting in the turnover of PtdIns(4)P at these sites.

## Supporting Information

Figure S1 **The 505-DELE-508 sequence adjacent to TM1 is not required for ER export.** HeLa cells were transfected with GFP-SAC1(478–549)-E2A (green), costained with anti-GM130 antibodies (red) and analyzed by confocal immunofluorescence microscopy. Scale bar, 50 µm.
(TIF)

Figure S2 **Replacing TM1 with the transmembrane domain of TfR2 results in loss of Golgi retention.** HeLa cells were transfected with the indicated GFP-tagged SAC1 constructs (green), costained with anti-GM130 antibodies (red) and analyzed by confocal immunofluorescence microscopy. (**A**) GFP-SAC1(478–549); (**B**) SAC1(478–549)TMtfr2. Scale bar, 50 µm.
(TIF)
