# Supplementary figures and images for: The First Transmembrane Domain of Lipid Phosphatase SAC1 Promotes Golgi Localization
Source: PLoS One. 2013 Aug 1;8(8):e71112. doi: 10.1371/journal.pone.0071112 (PMC3731292; doi:10.1371/journal.pone.0071112)

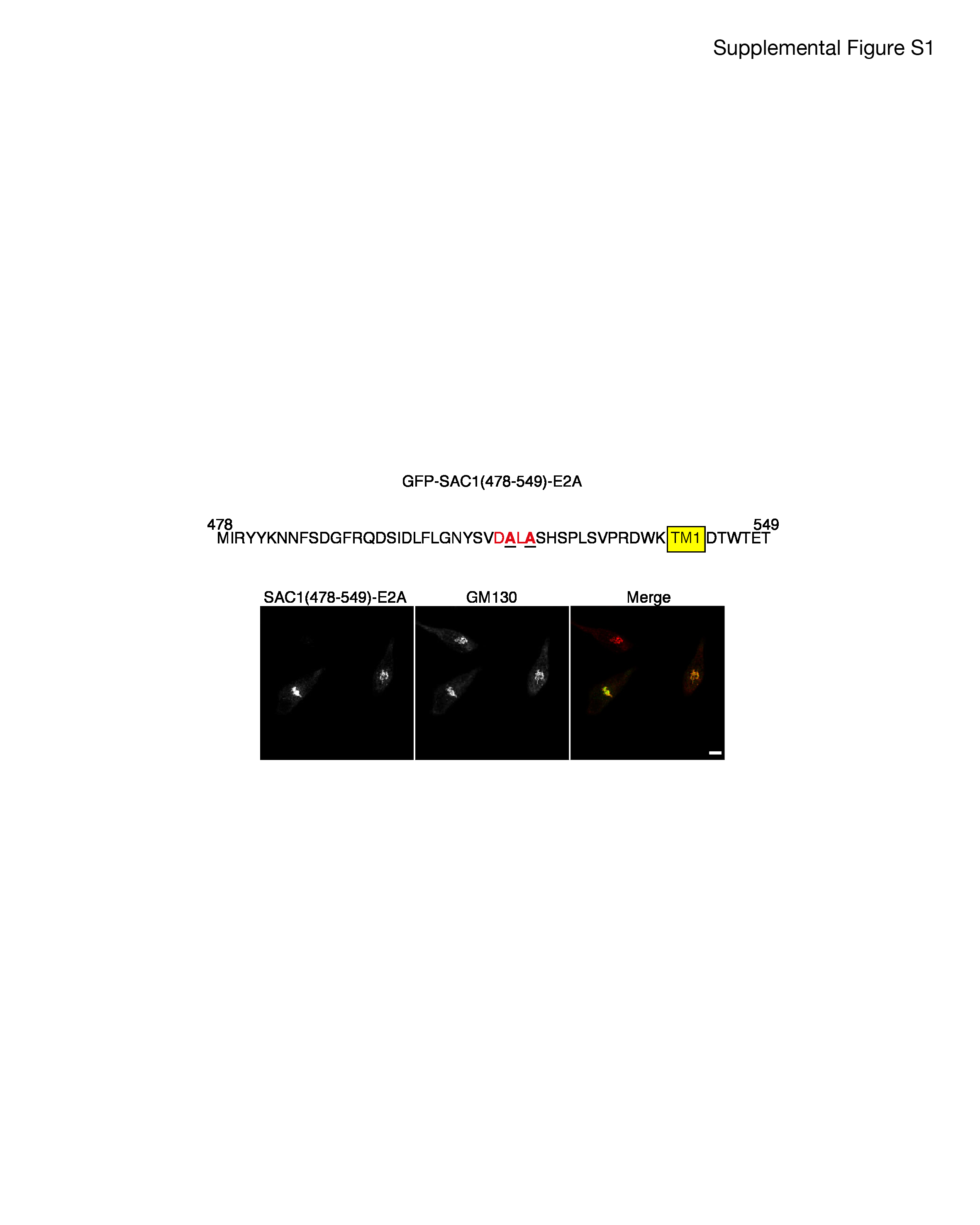

Supplement: Figure S1 — The 505-DELE-508 sequence adjacent to TM1 is not required for ER export. HeLa cells were transfected with GFP-SAC1(478–549)-E2A (green), costained with anti-GM130 antibodies (red) and analyzed by confocal immunofluorescence microscopy. Scale bar, 50 µm. (TIF) [file pone.0071112.s001.tif]

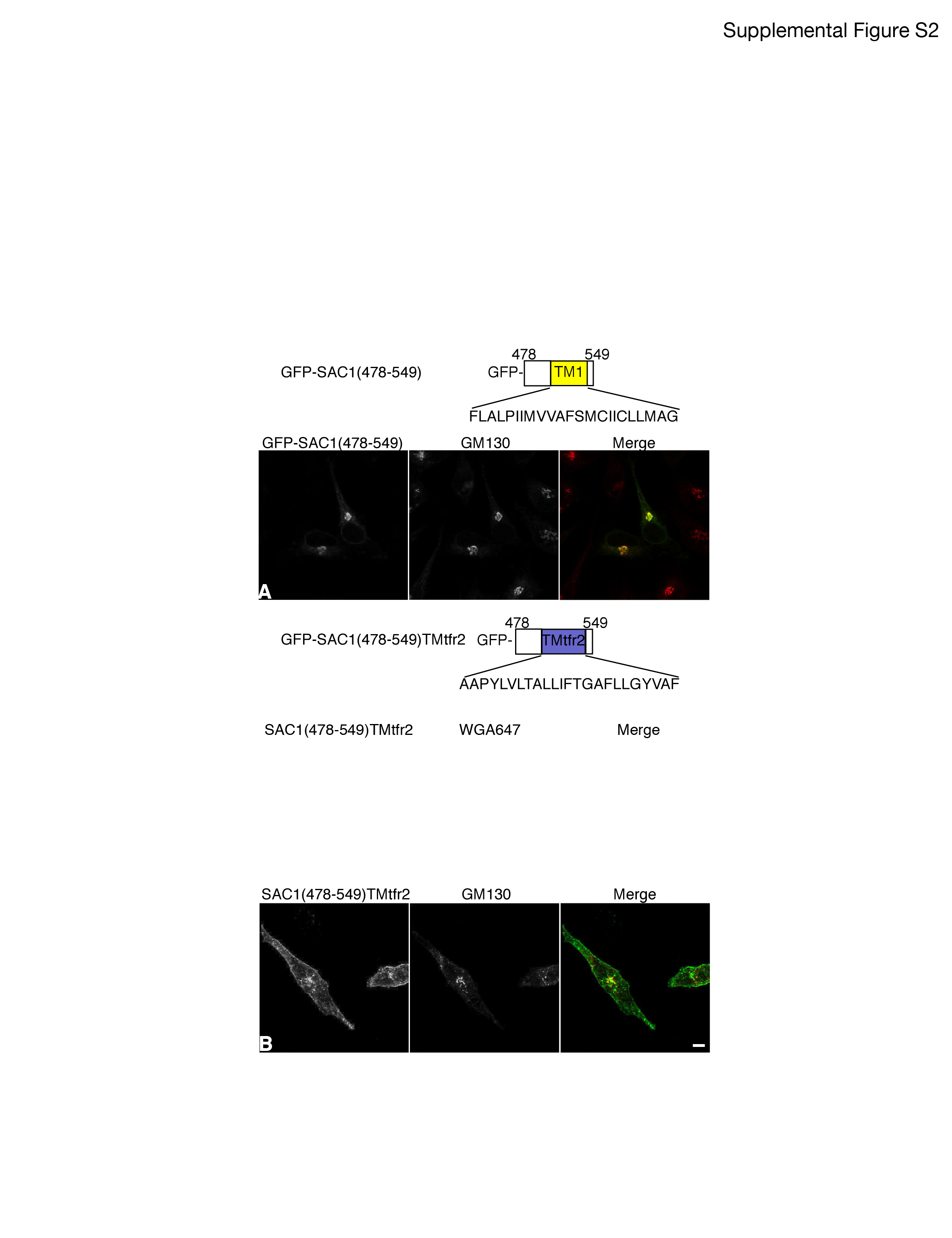

Supplement: Figure S2 — Replacing TM1 with the transmembrane domain of TfR2 results in loss of Golgi retention. HeLa cells were transfected with the indicated GFP-tagged SAC1 constructs (green), costained with anti-GM130 antibodies (red) and analyzed by confocal immunofluorescence microscopy. (A) GFP-SAC1(478–549); (B) SAC1(478–549)TMtfr2. Scale bar, 50 µm. (TIF) [file pone.0071112.s002.tif]
